# De novo transcriptome assemblies of C_3_ and C_4_ non-model grass species reveal key differences in leaf development

**DOI:** 10.1186/s12864-022-08995-7

**Published:** 2023-02-06

**Authors:** Santiago Prochetto, Anthony J. Studer, Renata Reinheimer

**Affiliations:** 1 Instituto de Agrobiotecnología del Litoral, Universidad Nacional del Litoral, CONICET, CCT-Santa Fe, Ruta Nacional N° 168 Km 0, s/n, Paraje el Pozo, Santa Fe, Argentina; 2 Department of Crop Sciences, University of Illinois, 1201 West Gregory Drive, Edward R. Madigan Laboratory #289, Urbana, IL 61801 USA; 3 Instituto de Agrobiotecnología del Litoral, Universidad Nacional del Litoral, FCA, CONICET, CCT-Santa Fe, Ruta Nacional N° 168 Km 0, s/n, Paraje el Pozo, Santa Fe, Argentina

**Keywords:** C_4_-photosynthesis, Proto-Kranz, Transcriptomics, Grasses, Otachyriinae, Differentially expressed genes, Differentially expressed orthogroups

## Abstract

**Background:**

C_4_ photosynthesis is a mechanism that plants have evolved to reduce the rate of photorespiration during the carbon fixation process. The C_4_ pathway allows plants to adapt to high temperatures and light while more efficiently using resources, such as water and nitrogen. Despite decades of studies, the evolution of the C_4_ pathway from a C_3_ ancestor remains a biological enigma. Interestingly, species with C_3_-C_4_ intermediates photosynthesis are usually found closely related to the C_4_ lineages. Indeed, current models indicate that the assembly of C_4_ photosynthesis was a gradual process that included the relocalization of photorespiratory enzymes, and the establishment of intermediate photosynthesis subtypes. More than a third of the C_4_ origins occurred within the grass family (Poaceae). In particular, the Otachyriinae subtribe (Paspaleae tribe) includes 35 American species from C_3_, C_4_, and intermediates taxa making it an interesting lineage to answer questions about the evolution of photosynthesis.

**Results:**

To explore the molecular mechanisms that underpin the evolution of C_4_ photosynthesis, the transcriptomic dynamics along four different leaf segments, that capture different stages of development, were compared among Otachyriinae non-model species. For this, leaf transcriptomes were sequenced, de novo assembled, and annotated. Gene expression patterns of key pathways along the leaf segments showed distinct differences between photosynthetic subtypes. In addition, genes associated with photorespiration and the C_4_ cycle were differentially expressed between C_4_ and C_3_ species, but their expression patterns were well preserved throughout leaf development.

**Conclusions:**

New, high-confidence, protein-coding leaf transcriptomes were generated using high-throughput short-read sequencing. These transcriptomes expand what is currently known about gene expression in leaves of non-model grass species. We found conserved expression patterns of C_4_ cycle and photorespiratory genes among C_3_, intermediate, and C_4_ species, suggesting a prerequisite for the evolution of C_4_ photosynthesis. This dataset represents a valuable contribution to the existing genomic resources and provides new tools for future investigation of photosynthesis evolution.

**Supplementary Information:**

The online version contains supplementary material available at 10.1186/s12864-022-08995-7.

## Background

C_4_ photosynthesis repeatedly, and in some cases rapidly, evolved as a mechanism that reduces the rate of photosynthesis in some environments (reviewed by [1, 2]). This adaptation became increasingly more advantageous in the past 30 million years, as CO_2_ levels declined and O_2_ levels increased in the atmosphere [3]. Although only about 3% of all angiosperms use the C_4_ pathway, C_4_ species are among the most economically important crops of the world (maize, sugar cane, sorghum, and millet), and C_4_ grasslands cover nearly 25% of the earth’s land surface [4–6]. Thus, C_4_ plants have a disproportionate influence on natural ecosystems and the world’s food supply.

Despite decades of studies, there are still many open questions about the genetic changes that occurred during evolution of C_4_ photosynthesis from a C_3_ ancestor. The C_4_ pathway requires changes in plant anatomy, cell structure, biochemistry, and physiology, and appears to involve dozens of genes. The leading hypothesis suggests that the assembly of C_4_ photosynthesis requires a series of evolutionary changes, which includes the relocalization of photorespiratory enzymes and the establishment of the intermediate C_2_ pathway [3, 7–9]. The C_2_ pathway represents a low-efficiency version of a photosynthetic carbon concentration mechanism in which the two-carbon compound glycine serves as a transport metabolite. Indeed, the C_2_ pathway is thought to be a major driver to the evolution of C_4_ photosynthesis because it initiates a shuttle of metabolites between the mesophyll and bundle sheath cells.

Surprisingly, the C_4_ pathway has evolved independently nearly 70 times in the angiosperms, with more than a third of the origins occurring in the grass family (Poaceae) [3, 10, 11]. Interestingly, few C_3_-C_4_ intermediate grass species do exist. In particular, the Paspaleae subtribe Otachyriinae presents a unique opportunity to investigate the origins of C_4_ photosynthesis in grasses. The Otachyriinae lineage includes 35 species distributed in the Americas, and has been the subject of recent phylogenetic and taxonomic work [12–14] (Additional file 1: Fig. S1). The current phylogeny shows multiple independent origins of species with intermediate anatomical and physiological characteristics that reduce levels of photorespiration (C_3_ Proto-Kranz [PK], and C_2_ photosynthesis), and at least one origin of C_4_ photosynthesis. The photosynthetic diversity present at Otachyriinae make this group a useful model to study the evolution of photosynthesis in closely related species.

Recent advancements in high-throughput sequencing technologies have enabled the use of genomic approaches to study the evolution of photosynthesis and the transcriptomic dynamics along a leaf development in model grass species [15–19]. Such results are an exceptional framework to advance in the understanding of photosynthesis on non-model species. In this work, we sought to investigate leaf gene expression of C_3_ (*Hymenachne amplexicaulis*), PK (*Rugoloa pilosa*), and C_4_ (*Antaenanthia lanata*) species of Otachyriinae subtribe from an evolutionary perspective. For each species, transcriptomes of four leaf segments, that capture different moments of development, were sequenced. Based on that, de novo assemblies were generated and annotated. Then, transcriptional profiles were comparatively investigated among segments and species.

## Results

### Leaf maturation in selected Otachyriinae species

**Fig. 1:**
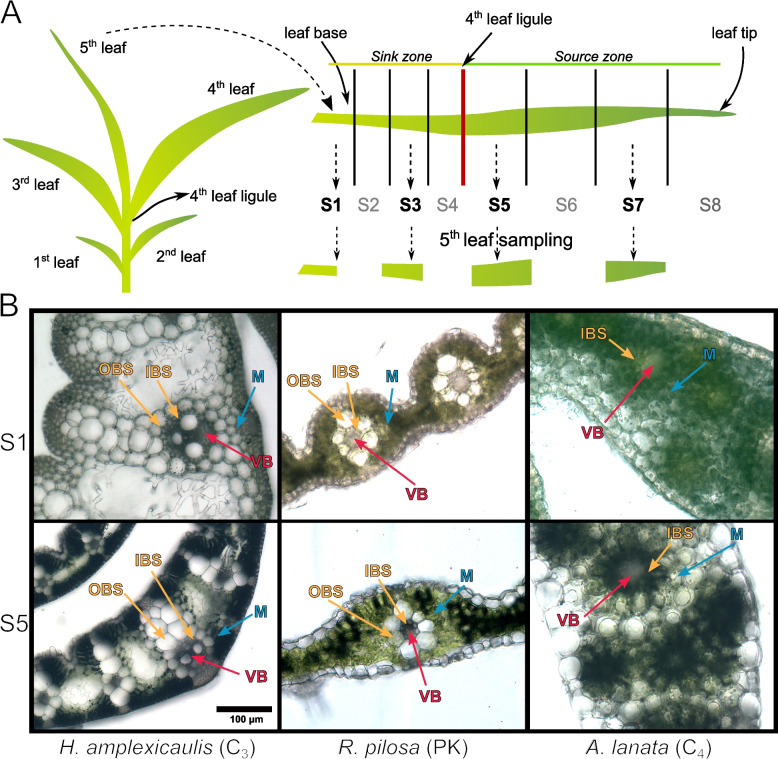
Sampling the 5th leaf of three Otachyriinae subtribe species. **A** Ligule from the 4th leaf was used as a marker to define the sink-source transition zone. Each zone was divided in 4 segments of equal length and the resulting 8 segments were labeled S1 to S8 from the base to the tip. Segments 1, 3, 5 and 7 were used for cross section cuts and transcriptomic assays. **B** Light microscope photographs of S1 and S5 cross section from C_3_ *H. amplexicaulis*, PK *R. pilosa*, and C_4_ *A. lanata*

Four different leaf cross sections that capture different moments of development of C_3_, *Hymenachne amplexicaulis* (Rudge) Nees*;* PK, *Rugoloa pilosa* (Sw.) Zuloaga; C_4_*, Anthaenantia lanata* (Kunth) Benth [14] were studied (Fig. 1a). Using light microscopy we observed that leaf segments of both C_3_ and PK species presented a typical C_3_ grass leaf anatomy with horizontally extended primary and secondary vascular bundles (VB) surrounded by two layers of bundle sheath cells without chloroplasts: the smaller Inner Bundle Sheath or mestome sheath (IBS) and the larger Outer Bundle Sheath (OBS) (Fig. 1b). The OBS of consecutive VB are separated by more than two mesophyll cell (M) layers. In particular, in *H. amplexicaulis,* we observed the presence of aerenchymatous tissue between VB as an adaptation to wetlands. By contrast, leaf segments of the C_4_ species *A. lanata* presented a tri-dimensional pattern of distribution of secondary VB. One layer of larger bundle sheath cells with chloroplasts, conventionally named IBS, surrounds both primary and secondary VB (Fig. 1b). In addition, one or two mesophyll cell layers separate the IBS from VB in *A. lanata* (Fig. 1b). Under light microscopy, we observed that segments 1 and 2 (S1, S2; leaf base) of each species are underdeveloped (in terms of present and distribution of chloroplast and vein development) in comparison with segments 5 and 7 (S5, S7; leaf blade) (Additional file 1: Fig. S2).

### Transcriptome assembly

The datasets generated and analyzed during the current study are available at NCBI Sequence Read with the accession N° PRJNA813546 (https://dataview.ncbi.nlm.nih.gov/object/PRJNA813546?reviewer=6vmhgdsbsobv6pd6u6tr9rp4oe). De novo transcriptome assembly was carried out using the Trinity package [20, 21]. Many transcript isoforms were detected after assembly. Indeed, we found 55.9, 136.9, and 112.2% more transcripts than genes for the C_3_, PK and C_4_ transcriptomes respectively (Additional file 1: TableS1). Although a high number of isoforms may be a possibility due to the complexity of grass genomes, lowly expressed isoforms could represent chimeric transcripts generated by the assembler. Therefore, only the most expressed isoforms were kept for downstream analyses. Coding sequences were identified using Transdecoder after redundant sequences were removed with CD-Hit [21, 22]. Transcript assemblies were then filtered by selecting the “single best” open reading frame (ORF) per transcript using Transdecoder [21] (Table 1; Additional file 1: Table S1).

**Table 1:** Assembly statistics for the C_3_ *H. amplexicaulis*, PK *R. pilosa* and C_4_ *A. lanata* de novo transcriptome assemblies

	***H. amplexicaulis***	***R. pilosa***	***A. lanata***
**N° of protein coding transcripts**	56,064	29,370	50,890
**Mean length**	911.9	1029.5	787.7
**Contig N50**	1233	1377	1059
**SALMON mapping rate [%]**	62.7	61.6	55.9
**BUSCO**	**C:85.7%**[S:83.9%,**D:1.8%**],F:3.3%,M:11.0%	**C:83.4%**[S:81.7%,**D:1.7%**],F:4.1%,M:12.5%	**C:82.8%**[S:81.1%,**D:1.7%**],F:4.5%,M:12.7%

To evaluate the level of duplicate sequences and transcriptome completeness, a BUSCO search against the monocots database was performed [23] (Table 1). The results showed a high level of completeness for the de novo transcriptome assemblies of each species (85.7, 83.4 and 82.8% for C_3_, PK and C_4_ species respectively), and a low level of duplicated sequences (1.8, 1.7 and 1.8% for C_3_, PK and C_4_ species respectively) for all transcriptomes. Quality information for the final transcriptomes is shown in Table 1. The total number of protein coding transcripts varied widely between species: 56,064 transcripts for C_3_ *H. amplexicaulis*, 29,370 for PK *R. pilosa,* and 50,890 for C_4_ *A. lanata*.

### Transcript annotation and ortholog inference

**Fig. 2:**
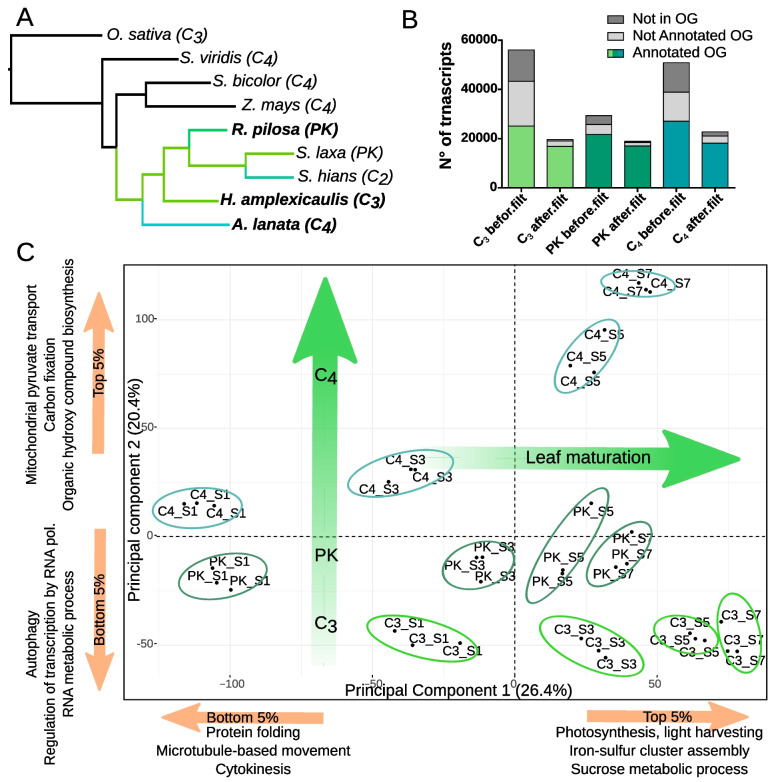
Exploratory analysis of the transcriptomes. **A** Species rooted tree based in single copy orthologs, generated with Orthofinder. **B** Transcriptome size before and after filtering low expressed transcripts. **C** Principal components analysis of the samples. Enriched GO terms with the highest load in each dimension are indicated in the corresponding axis.

For transcript annotation and ortholog identification, Orthofinder was used with six reference proteomes from Otachyriinae subtribe species *Steinchisma hians*, *Steinchisma laxa*, and four grass outgroups, *Zea mays*, *Sorghum bicolor*, *Setaria viridis* and *Oryza sativa*. The species tree reconstructed by orthofinder is consistent with the species phylogeny reported in the literature (Fig. 2a; Additional file 1: Fig. S1). Around 81% of transcripts were placed in orthogroups (OG) and 2246 single-copy OG were detected between the nine transcriptomes. When *S. bicolor* sequences were present in an OG we used *S. bicolor* sequence information to infer the annotation for the Otachyriinae transcripts. In the OG where *S. bicolor* sequences were absent, we used annotations from *Z. mays* or *S. viridis*. A total of 25,134 (44.8%), 21,710 (73.9%), and 27,147 (53.3%) transcripts were annotated for C_3_ *H. amplexicaulis*, PK *R. pilosa* and C_4_ *A. lanata* respectively (Fig. 2b; Additional file 1: Table S2). Despite the differences in transcriptome sizes, the number of annotated transcripts were similar. To further explore the annotation rate differences, transcripts were filtered based on expression (less than 1 cpm in 3 replicate libraries), and the proportion assigned to OG was assessed. The results showed that 91.6, 90.3, and 79.7% of the transcripts that were retained after filtering LET were annotated transcripts belonging to OG for C_3_, PK and C_4_ respectively (Fig. 2b; Additional file 1: Table S2). Altogether, this suggests that transcripts annotated from OG represent a core set of high-quality transcripts.

A Principal Component Analysis (PCA) of samples, using expression values from 13,373 OG present in all three species, showed replicates grouping together (Fig. 2c). We observed that PC1 explains 26.4% of the variation and discriminates samples by developmental stage and PC2 explains 20.4% of variation and discriminates samples by species. In order to understand the biological processes represented by the principal components, we calculated the load factors of each OG for both components, and identified gene ontology terms enriched in the OG with the top and bottom 5% load factors [24] (Additional File 2: Supplementary datasheet 1). Results indicated that PC1 represented processes related to cellular development such as protein folding and transport, cytokinesis, photosynthesis and sucrose metabolism. The load factors from PC2 were negatively associated with autophagy, regulation of transcription and RNA metabolism. Positively associated terms were related to C4 metabolism such as mitochondria pyruvate transport, carbon fixation and organic hidroxy compound biosynthesis. We also found that PC3 discriminates PK samples from other species explaining 15.9% of variation and PC4 correlated with sink-source transition zone samples and explains 10% of variance (Additional File 1: Fig. S3). 

### Differentially expressed genes

#### Defining sink and sources zones

To link our anatomical observations with gene expression along the species leaf segments, we investigated the transcript levels of genes that are known markers of sink and source zones [15, 16, 25, 26]. Sink markers, such as cell cycle modulators Cyclin (CycD4 and CycD6) and sucrose synthase (SUS), decrease in expression from S1-S3 to S5-S7, indicating that S1 and S3 belong to the leaf sink zone. By contrast, the expression level of the source markers sucrose transporters (SWEET and STP1) and nitrate reductase (NR1) are low in segment S1 and S3 but have increased expression in S5 and S7, indicating that S5 and S7 correspond to the source zone of the leaves (Additional file 1: Fig. S4).

#### Transcriptome dynamics of developing leaves

**Fig. 3:**
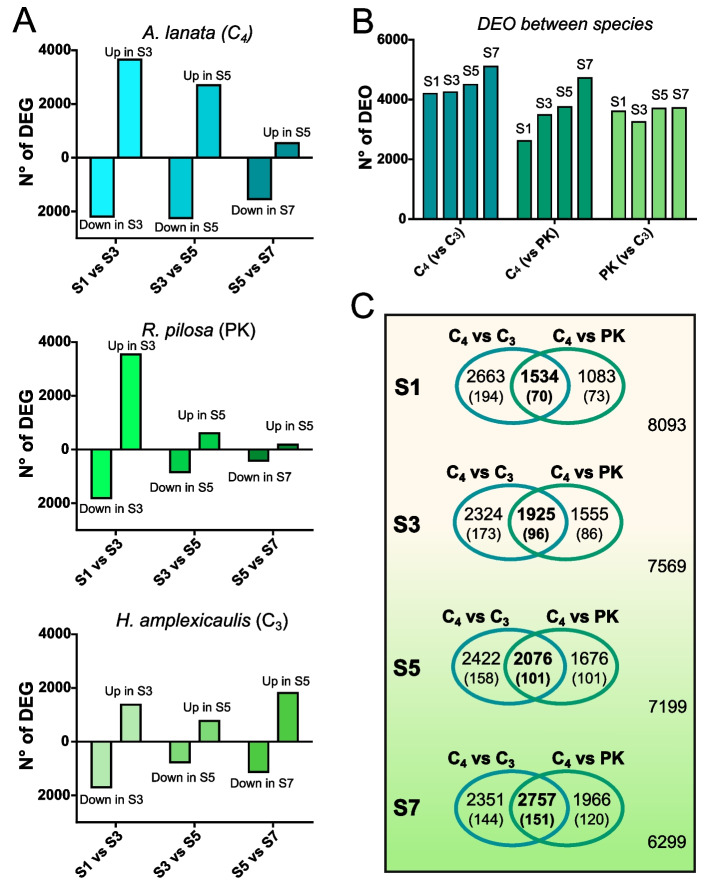
Intraspecies and cross species differential expression analysis. **A** Number of differentially expressed genes (DEG) between leaf segments in *A. lanata* (C_4_), *R. pilosa* (PK) and *H. amplexicaulis* (C_3_). **B** Number of differentially expressed orthogroups (DEO) between leaf segments of different species. **C** Venn’s diagrams showing the number of DEO between C_4_ species and the other two C_3_ species, across leaf segments. Numbers between brackets correspond to DEO that are annotated as transcription factors

We studied the dynamics of the transcriptomes along the leaf development for each of the three species. Pairwise comparisons within species were done between consecutive segments to evaluate the number of differentially expressed genes (DEG) (Fig. 3a). The transition between S1 and S3 resulted in the most DEG in all species, and the number of DEG tends to decrease during leaf maturation. Overall, when the number of DEG in adjacent segments were compared between species, we observed that the C_3_ leaf showed a lower number of DEG than the C_4_ leaf. The PK presented intermediate numbers of DEG, similar to the C_4_ maximum in S1-S3 transitions and the C_3_ minimum in S3-S5 and S5-S7 transitions. These results imply a more dynamic leaf transcriptome in C_4_ species in terms of the amount of DEG along the leaf segments. In terms of total DEG, we observed that the PK transcriptomic profile resembles that of the C_4_ leaf more than that of the C_3_ for every leaf segment (Fig. 3b).

A Gene Ontology (GO) analysis was performed with TopGO to obtain information about the biological processes enriched in the DEG in each transition. Overall, the three species showed a well-preserved developmental progression, with differences in the onset and length of several processes (Additional file 3: Supplementary datasheet 2). All species showed GO terms related to photosynthesis (such as light harvesting, photosystems I and II, thylakoid membrane, etc.), enriched in upregulated DEG in S3 compared to S1. In particular, the C_4_ species, GO terms related to photosynthesis continue to be present in the upregulated DEG of S5 compared to S3. All species showed GO terms associated with cell growth and development (DNA replication, modification of the cell wall, development and cellular division processes) in down regulated DEG in S3 compared to S1. In addition, the C_4_ species included terms related to translation and ribosome biogenesis in S3 compared to S1 (Additional file 3: Supplementary datasheet 2). In the later stages of leaf development, comparisons between S3 and S5 of all species showed DEG enriched in the categories of oxide-reduction, carbohydrate metabolism, and response to oxidative stress. In particular, in S5 of C_3_*,* transcripts involved in protein phosphorylation and defense response processes were upregulated. Finally, in all species, terms such as cellulose biosynthetic process, cell wall modification, and transmembrane transport were detected late in development, both in comparisons between S3 and S5 as well as S5 and S7 (Additional file 3: Supplementary datasheet 2).

#### Gene expression comparisons at successive stages of leaf development between species

Between species, comparisons of differential gene expression among segments of the same developmental stage were analyzed using orthologs inferred from Orthofinder (Fig. 3b and c). In most cases OG were made of single copy orthologs (70.3, 78.8 and 62.8% in C_4_, PK and C_3_, respectively). In the case of multicopy OG, the sum of the expression values of the transcripts was used and summarized the annotation information. As a result, 13,373 OG were tested to find Differentially Expressed OG (DEO).

The number of DEO between species were 8264, 7087, and 6518 for C_4_-C_3_, C_4_-PK and PK-C_3_, respectively. The proportion of TF within these DEO (6.6, 6.1, and 6.6% respectively for each comparison) is in line with the overall proportion of TF in the total of OG studied (6.3%, or 851 out of 13,373 OG). Overall, the number of DEO between C_4_ and the other species increased from base (immature sink segments) to tip (mature source segments). In contrast, the number of DEO between C_3_ and PK remains relatively constant across segments (Fig. 3b). The number of DEO between C_4_ and PK showed a stepwise increase from immature (S1, 2617 DEO) to mature segments (S7, 4723 DEO). However, the number of DEO between C_4_ and C_3_ showed a substantial difference in S1 (4197 DEO), which increases only moderately in mature segments (S7, 5108 DEO) (Fig. 3c). In terms of annotated transcription factors, we observed that the number of transcription factors belonging to DEO between C_4_ and the other species, doubles from base to tip (70 to 151 from S1 to S7) when species are compared. Meanwhile, the proportion of TF to total DEO has a soft increase from 4.5% (70/1534) to 5.5% (151/2757) between S1 and S7) (Fig. 3c).

#### Differentially expressed orthogroups involved in photosynthetic processes along leaf development

**Fig. 4:**
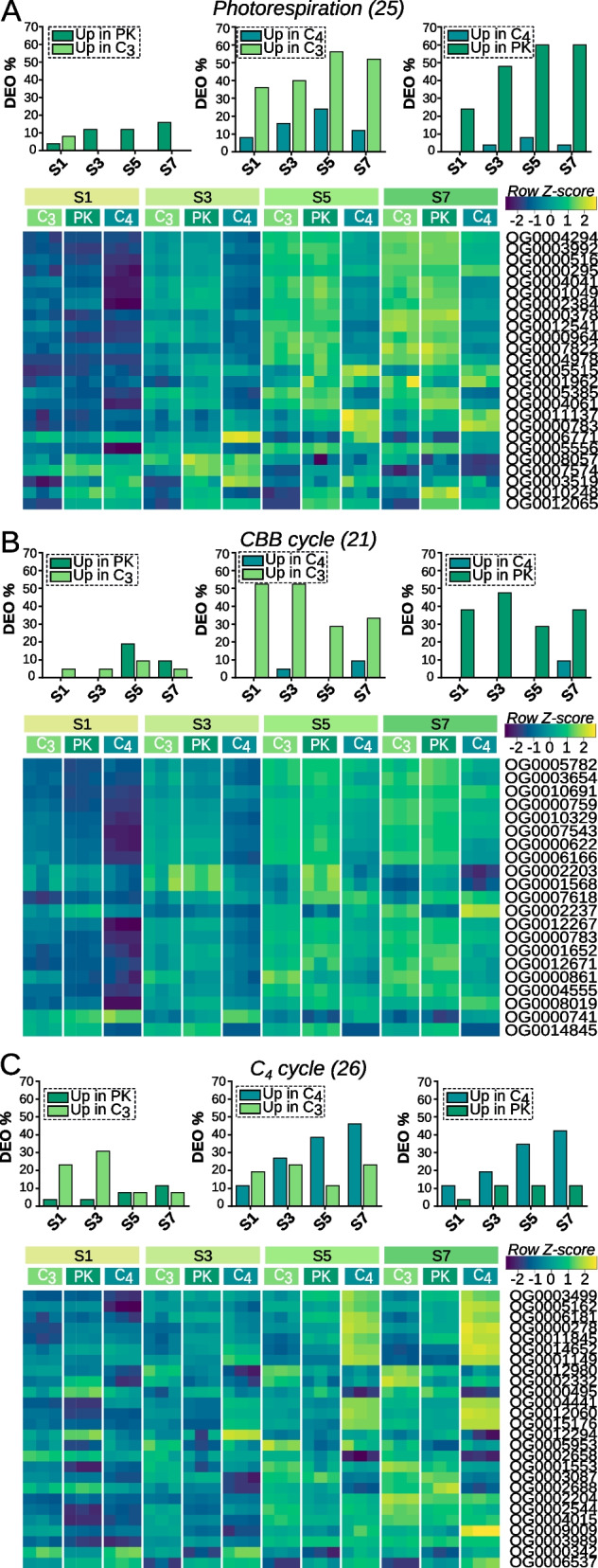
Percentage of DEO between species and heatmaps showing photosynthesis related OG expression across leaf segments. **A** Photorespiration OG. **B** CBB cycle OG. **C** C_4_ cycle OG

To study the processes related to photosynthesis, OG lists were constructed according to GO terms and genes classified in previous studies [27–29] (Additional File 4: Supplementary datasheet 3). For each list, the percentage of DEO between species was estimated according to the stage of development (Fig. 4). In terms of DEO, the differences between C_3_ and PK species were smaller than those observed between these species and the C_4_. We observed differences in OG related to photorespiration and Calvin-Benson-Bassham cycle (CBB cycle) from base to tip between C_4_ species and PK and C_3_ species (Fig. 4A-B). In particular, 36% and 24% of the OG involved in photorespiration were upregulated in S1 of C_3_ and PK, respectively. Similarly, 52% and 38% of the OG included in the CBB cycle were upregulated in S1 of C_3_ and PK, respectively. Overall, the proportion of DEO related to photorespiration increased from base to tip between C4 and the other species; however, DEO related to CBB decreased in source segments. The number of upregulated OG in the C_4_ cycle group increases from base to tip for the C_4_ species, reaching 46.2% and 42.3% in S7 for C_4_ vs C_3_ and C_4_ vs PK respectively (Fig. 4c). Also, we observed that, as leaves mature, the differences in expression in the C_4_ cycle between C_4_ and C_3_ species are slightly higher than compared to PK species.

Additionally, a heatmap is presented for each group showing the relative expression of each OG along the leaf segments and by species (Fig. 4; Additional File 1: Fig. S5A-C). We observed that: (1) most OG present an ascending pattern of expression from base to tip and, (2) the differences in the relative expression of each OG between C_4_ and the other species tend to be magnified, also, from base to tip. Overall, these results may suggest that photosynthetic machinery is activated later in leaf development of the C_4_ than in the C_3_ and PK species.

#### Differential expression of orthogroups in vasculature regulation and suberin biosynthesis processes along leaf development

**Fig. 5:**
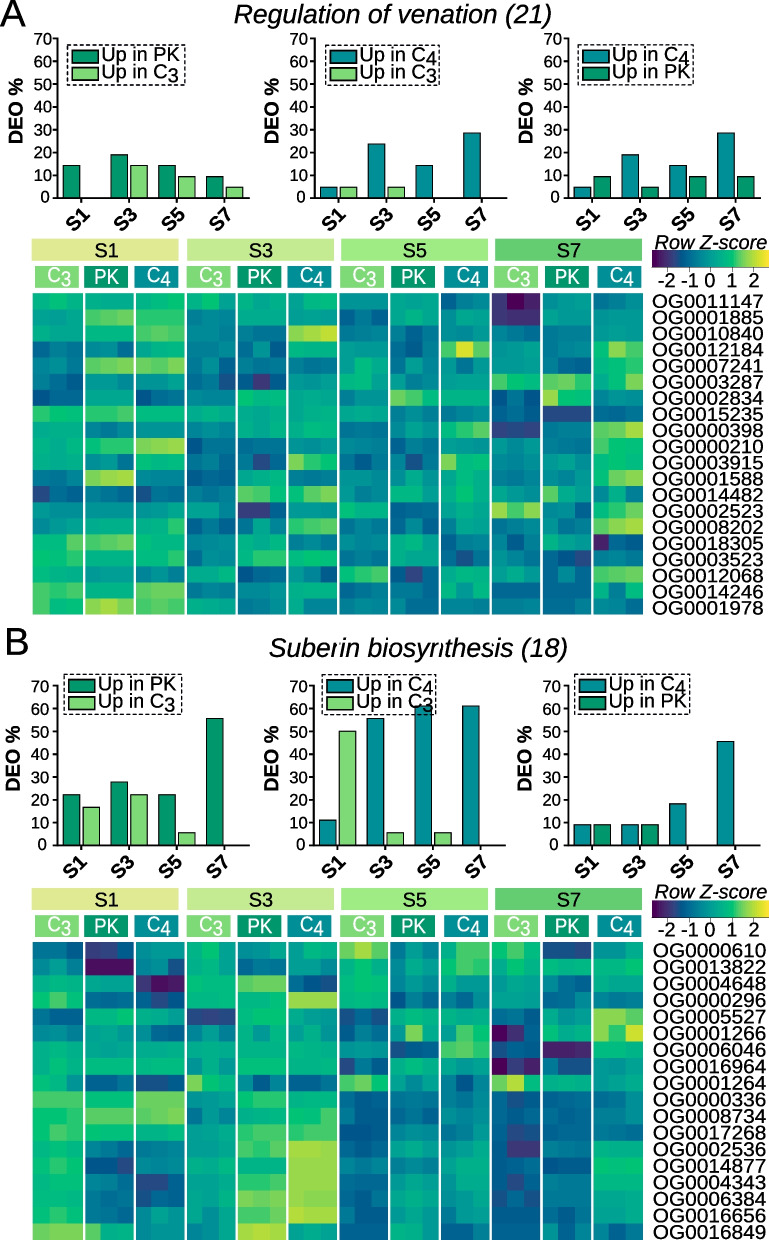
Percentage of DEO between species and heatmaps showing developmental related OG expression across leaf segments. **A** regulation of venation OG. **B** Suberin biosynthesis OG

Our results documented a delay in the expression of transcripts related to photosynthesis of the C_4_ leaf in comparison to leaves of C_3_ and PK species. To investigate whether the delay correlates with the existence of a longer period of anatomical development that precedes photosynthesis maturation, we looked for markers of leaf anatomical development. In particular, we analyzed the expression of genes related to vascular bundle formation and suberin biosynthesis (Additional file 4: Supplementary datasheet 3). Indeed, OG related to the regulation of venation were upregulated in C_4_ species compared to C_3_ and PK species (Fig. 5a). This is particularly evident from S3 to S7, in line with the ascendant pattern of vascular density in the C_4_ leaf during development. Based on the heat maps for these OG, we noted that many OG related to regulation of venation are highly expressed at the leaf base (S1) of C_3_ and PK species and to a lesser extent in C_4_ species (Fig. 5a; Additional File 1: Fig. S5D). Subsequently, OG expression declines rapidly in C_3_ species, whereas in PK species this decline appears to be more gradual (Fig. 5a). On the other hand, we observed an increase in OG expression in S7 of C_4_ species (Fig. 5a).

We also observed contrasting patterns of expression when suberin biosynthesis OG were compared between C_4_ and C_3_/PK species (Fig. 5b; Additional File 1: Fig. S5E). While suberin biosynthesis OG are expressed at S1 in C_3_ species, in PK and C_4_ species the maximum of expression is reached in S3 for most of the OG.

In vasculature regulation and suberin biosynthesis processes, we observed a shift of DEO from S3 to S7 when C_3_/PK and C_4_ were compared. This may suggest that these processes are activated early (S1) in leaf development of C_3_/PK species, while they are turned on later (S3-S7) in C_4_ leaf species.

#### Expression patterns and levels of expression for C_4_ cycle and photorespiration OG

**Fig. 6:**
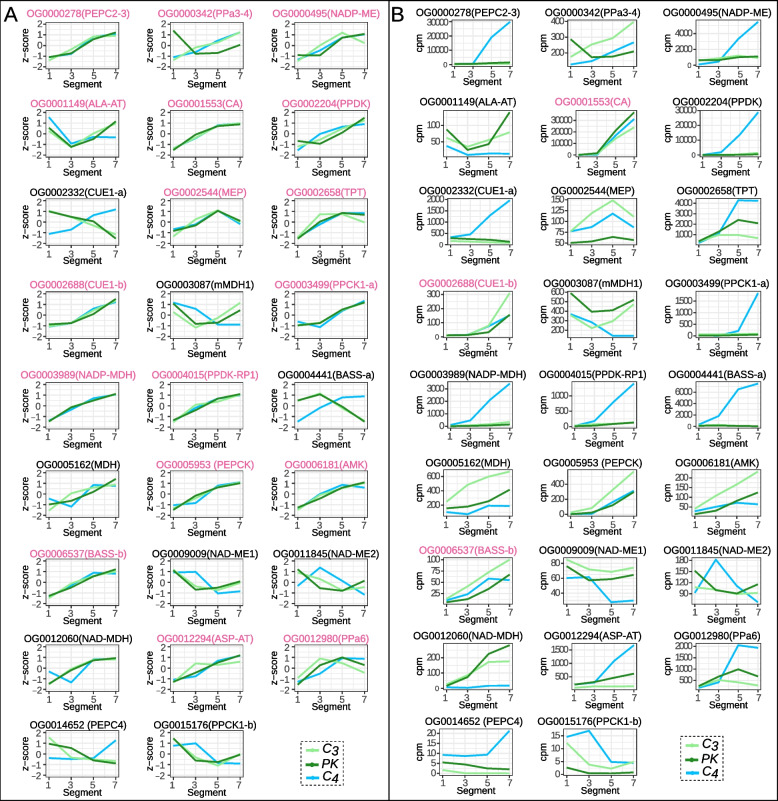
Expression patterns for C_4_ cycle related OG. OG mean expression values from S1, 3, 5 and 7 for C_3_-*H. amplexicaulis* (light green), PK-*R. pilosa* (dark green) and C_4_-*A. lanata* (light blue). **A** Relative expression (z-score normalization) across segments. **B** Count per million (cpm) across segments. . Names in pink indicate OG with conserved expression patterns between the C_4_ species and at least one of the C_3_/PK species. Abbreviations: PEP carboxylase (PEPC2–3, PEPC4), PPDK associated (PPa3–4, PPa6), NADP Malic enzyme (NADPME), alanine aminotransferase (ALA-AT), carbonic anhydrase (CA), pyruvate phosphate dikinase (PPDK), CAB underexpressed 1 (CUE1-a, CUE1-b), mesophyll envelope protein (MEP), triose phosphate translocator (TPT), mitochondrial malate dehydrogenase (mMDH), phosphoenolpyruvate carboxylase kinase 1 (PPCK1-a, PPCK1-b), NADP malate dehydrogenase (NADP-MDH), PPDK regulatory protein 1 (PPDK-RP1), bile acid sodium symporter (BASS-a, BASS-b), malate dehydrogenase (MDH), phosphoenolpyruvate carboxykinase (PEPCK), AMP kinase (AMK), NAD malic enzyme (NAD-ME1, NAD-ME 2), NAD malate dehydrogenase (NAD-MDH), aspartate aminotransferase (ASP-AT)

To investigate the dynamic regulation signatures of the C_4_ cycle, the expression patterns and transcript levels of OG that encode for enzymes and transporters in the C_4_ pathway were compared across the leaf segments (Fig. 6). In order to be able to compare the expression patterns across species, a *z*-score normalization of the OG expression was performed individually. We found that in 17 of the 26 OG related to the C_4_ cycle, the expression pattern along the leaf segments were preserved among species despite having different photosynthetic pathways (Fig. 6a). Overall, most of the key enzymes of the C_4_ cycle show an ascending pattern as the leaves develop.

Although the expression patterns were preserved for most of OG, the levels of expression varied depending on the species and leaf segment analyzed. Meanwhile only three OG showed similar levels of expression in all species (CA, CUE1-b and BASS-b); the expression of most of the OG was substantially higher in C_4_ mature segments (Fig. 6b). The expression level of the OG associated with PEP-CK and AMK were similar in the C_4_ and PK species, but higher in the C_3_ species (Fig. 6b). In mature segments, decarboxylating enzymes associated with the subtypes NAD-ME and PEP-CK are less expressed in C_4_ than in C_3_ species. The OG involved in photorespiration behaved similarly as the C4 cycle. All of the OG sampled here have a conserved expression pattern among species, but only a few had similar levels of expression (Additional file 1: Fig. S6 and Fig. S7).

## Discussion

Closely related non-model species with different carbon assimilation pathways represent an underutilized resource for understanding the genetic changes that drive photosynthetic evolution. Here we present high-confidence protein-coding leaf transcriptomes from three species in the Otachyriinae subtribe. Transcriptomes were assembled across four different leaf segments that capture different stages of development. This dataset represents a valuable contribution to the existing genomic resources and provides new tools for future investigation of photosynthesis evolution.

The de novo assembly of the transcriptomes contained a large number of genes and transcripts. These preliminary results seem inflated compared to estimates of gene and transcript numbers in other grass species [30]. The large number of genes and transcripts observed in our dataset compared with the known *Z. mays*, *S. bicolor*, and *S. viridis* transcriptomes may be explained by one or a combination of three possible phenomena: (a) the presence of a large number of the isoforms in our transcriptomes, (b) the product of sequencing errors or incomplete transcripts in poorly expressed genes and/or, (c) the presence of pre mRNA in highly expressed transcripts [31, 32]. Isoform filtering resulted in a reduction in the number of transcripts by approximately 50%, while the mapping rate had a minor reduction from 95 to 97% to 87–91%. This hypothesis, like many others related to the quality of transcriptomes, can be verified when the genomes of these species are sequenced.

Additionally, a substantial reduction in the number of genes was observed when sequences without a complete or partial ORF were filtered from the datasets. In this step, two-thirds of the C_3_ and C_4_ sequences and half of the PK sequences were removed. However, this resulted in a decrease in the mapping rate from 87 to 91% to 56–63%. Part of this reduction is likely due to the removal of UTR segments from transcripts by Transdecoder. Besides spurious transcript assemblies, filtered sequences may belong to non-coding RNA and transposable elements given that libraries represent enrichments of mRNA from a total RNA sample. Similar results were obtained when the maize leaf transcriptome was analyzed [15]. Indeed, Li et al. (2010) found that 84% of the reads mapped to protein-coding genes, while the rest belonged to introns, intergenic regions, transposable elements, and splice junctions between exons. Finally, we confirmed that most of the sequences that were removed because they were very poorly expressed did not belong to any OG. In addition, after filtering by expression values, both the sizes of the transcriptomes and the percentage of the annotated sequences were remarkably homogeneous (Additional file 1: Table S1 and S2).

The quality analysis of the transcriptomes performed with BUSCO found two interesting results. First, the number of duplicated sequences is very low (1.7 to 1.8%), which implies little redundancy in the transcriptomes. Second, the completeness of the de novo transcriptome assemblies was higher than expected given that they were derived from a single tissue type. When using only leaf tissue samples, it would be expected to find a limited number of transcripts involved in the germination processes, root development or flower development, resulting in moderate levels of completeness. Indeed, we found a relatively low number of missing sequences (11.0 to 12.7%). The high transcriptome completeness presented could be due to the variety of leaf developmental stages used. Alternatively, the total number of reads used in the assembly (around 420 million reads per species) increased our ability to detect lowly expressed transcripts.

### Divergent leaf transcriptome dynamics among species

An analysis of DEG found that immature segments showed a higher transcriptional dynamic, estimated as the number of DEG, whereas in the mature segments the dynamics substantially decreased. This pattern is observed in C_4_ and PK leaves; however, the trend of decreasing DEG is reduced in the C_3_ leaf segments. These results are in agreement with the leaf developmental gradients previously reported in several monocot species [15–17, 19].

Interestingly, among species studied in this work, only *A. lanata* maintains high levels of DEG until S5 (Fig. 3). Gene ontology analysis confirmed that genes involved in leaf anatomy differentiation are overrepresented until S5 suggesting a longer time of leaf differentiation in the C_4_ leaf in comparison with leaves of C_3_ and PK species. This result suggests that the C_4_ leaf takes more time to complete the necessary anatomical differentiation prior to the activation of the photosynthetic machinery. Thus, expression of photosynthetic genes is delayed to more mature segments of the leaf, in comparison with C_3_ and PK species. Interestingly, the longer time of leaf differentiation and the resultant delay in the activation of the photosynthesis machinery may be a peculiarity of the unique leaf anatomy of *A. lanata* as has also been shown for *Cleomaceae* [28]. In fact, *A. lanata* showed a tri-dimensional pattern of distribution of secondary VB in contrast with the horizontally extended primary and secondary VB of most of the grasses. Indeed, neither maize, sorghum, rice nor *Dichanthelium* presented such delay in the activation of the photosynthesis machinery [15, 17–19]. Notably, *R. pilosa* presents an intermediate development pattern between the two species, with an increase in the dynamics of expression in the first developmental transition and a marked decrease in the following transitions. Interestingly, phylogenetic studies in Otachyriinae place *H. amplexicaulis* and *R. pilosa* as close relatives but, in terms of DEG, the leaf transcriptome of PK is more similar to that of C_4_ in the immature segments of the leaf. This may suggest commonalities in the dynamic of the expression at the sink zone of PK and C_4_ leaves.

### Conserved expression pattern and diverse level of expression

To analyze the degree of conservation of C_4_ photosynthesis related processes, we analyzed the expression patterns of key OG. A conserved pattern was observed for enzymes associated with the NADP-ME subtype of the C_4_ pathway along the leaf segments for the C_3_, PK, and C_4_ studied species. Conservation of the expression patterns between C_3_ and C_4_ species have also been described in other taxonomic groups [33]. In particular, Xu et al. (2016) found that 7 out of a total of 15 genes that encode for C_4_ enzymes showed conversed expression patterns during the process of rice and corn leaf de-etiolation. Interestingly, we find 14 of the 15 genes previously studied by Xu et al. (2016) have highly conserved expression patterns along the leaf segments of the C_3_, PK, and C_4_ species studied here. This result suggests a higher degree of conservation of the C_4_ pathway in Otachyriinae, likely due to the evolutionary close proximity of the studied species.

Although we observed a uniform expression pattern in C_4_ OG among the different leaf types, we confirmed differences in the levels of expression of key photosynthetic enzymes and photorespiratory markers between the leaf of the C_4_ species and C_3_/PK species. Overall, the leaf of *A. lanata* showed that OG encoding for C_4_ enzymes are highly expressed in mature segments of the leaves compared to the studied C_3_/PK species. In addition, most of the OG that codes for photorespiratory pathway presented an ascending pattern of expression from base to tip for all species; however, the difference lies in the level of expression in the source segments, where more than 50% of them are up regulated in C_3_/PK vs C_4_ species.

In summary, transcriptome analysis shows the conservation of expression patterns of C_4_ cycle genes and photorespiratory cycle in C_3_/PK/C_4_ species suggesting that the developmental program precedes the evolution of C_4_ photosynthesis. In this scenario, installing new pathways in C_3_ species would require fewer changes in expression patterns during the development and an increase of the expression activity of key genes at the right moment of leaf development.

## Conclusion

New transcriptomes presented here expand the leaf development of closely related non-model grass species C_3_, intermediate and C_4_ leaves. We found commonalities and key differences on the leaf transcriptome performance among the three studied species as well as with other grass species. Overall, we found that genes associated with photorespiration and the C_4_ cycle are differentially expressed between C_4_ and C_3_ species, whereas their expression patterns are well preserved throughout leaf development. Indeed, similar trends were documented for rice and maize [31]. Interestingly, the C_3_-PK transcriptomic profile is more similar to C_4_ than C_3_ in early stages of development suggesting that *R. pilosa* and *A. lanata* have commonalities in the transcription activity during the anatomical setup but are different in the biochemistry of photosynthesis. Finally, the analysis of the transcriptomes showed some peculiarities of the gene expression along the leaf segments of *A. lanata* leaf, which may correlate with its unique foliar anatomy among grasses.

This dataset represents a valuable contribution to the existing genomic resources and provides new tools for future investigation of photosynthesis evolution.

## Methods

### Plant material and growth conditions

Plant specimens were deposited at “Arturo E. Ragonese” Herbarium (SF) (Facultad de Ingeniería Agronómica, Universidad Nacional del Litoral, Esperanza, Santa Fe, Argentina). The detailed voucher numbers are listed in Additional file 1 (Fig. S1). Individuals of *H. amplexicaulis* (C_3_), *R. pilosa* (C_3_-Proto Kranz) and *A. lanata* (C_4_- NADP-ME) were grown in a grow chamber at 27 °C under long day conditions (16 hours of light and 8 hours of darkness). For each species, the 5th young leaf of 90 individuals was collected when it reached the length of the 4th leaf. Each leaf was divided into two sections, taking the sink-source transition zone as a reference. The sections were then divided into four leaf segments of equal length and labeled S1 to S8 from the base to the tip of the leaf. For transcriptomic sequencing, segments 1, 3, 5 and 7 from 90 leaves were collected in 9 pools of 10 individuals each, which were randomized to create 3 replicates of 30 individuals. For histological analysis segments 1, 3, 5 and 7 from 10 leaves were collected. The replicates are paired samples.

### Histological analysis

Fresh leaf segments were arranged in molds with 5% low melting point agarose in PBS 0.05 M at 50 °C and left to harden at 4 °C for 1 hour. A Leica VT1000S (Leica Biosystems, Germany) vibratome was used to obtain 100 μm thick sections of plant material. Sections were mounted onto microscope slides and covered with PBS 0.05 M to be studied under light microscopy (Nikon Eclipse E200).

### Transcriptome sequencing and assembly

Total RNA was extracted with Tripure reagent (Sigma) following the manufacturer’s protocol. An additional purification of the RNA was carried out using LiCL precipitation [34]. Libraries were prepared by the Roy J. Carver Biotechnology Center at the University of Illinois using the standard Illumina TruSeq mRNA sample prep kit. Thirty-two libraries were pooled and sequenced using an Illumina NovaSeq SP flowcell lane that produced 22 to 45 million 250 bp paired-end reads per library.

The selected pipeline for de novo transcriptome assembly was based on Carruthers et al. (2018) and Moreno-Santillan et al. (2019) [35, 36] The raw read quality of each paired-end library was examined using the bioinformatics tool FastQC v0.11.5 [37]. Low quality reads were trimmed and filtered with trimmomatic  [38]. After this, between 3 and 7% of the reads were discarded. De novo transcriptome assembly was carried out using Trinity v.2.8.5 [21]. The proportion of reads mapped to the assembly was assessed with Bowtie2 v2.3.2  [39].

To reduce the probability of obtaining spurious transcripts and attenuate transcript redundancy, the contigs were filtered using three methods. First, weakly expressed isoforms were removed based on their expression values. For that, TPM values were obtained by SALMON v0.14.1 [40], and weakly expressed isoforms were removed using the Trinity script filter_low_expr_transcripts.pl with “–highest_iso_only” parameter. Second, a set of non-redundant representative transcripts was generated using the CD-Hit package v4.6.6 [22] with an identity threshold of 95%. Finally, transdecoder v5.1.0 [21] was used to identify all likely coding regions within our assembled transcripts, and then filtered by selecting the single best ORF per transcript. Any transcripts with ORFs less than 100 bp in length were removed before performing further analyses. Transcriptome completeness and redundancy was assessed using the bioinformatics tool BUSCO v.3.0.1 (Benchmarking Universal Single-Copy Orthologs) to obtain the percentage of single-copy orthologs represented in the monocots dataset [23].

### Gene annotation

Orthofinder v2.3.7 [41] was used for orthologues identification using six reference proteomes. Two transcriptomes were from the same subtribe, *Steinchisma hians* and *Steinchisma laxa* (Studer unpublished data), and four grass outgroups, *Zea mays RefGen_V4*, *Sorghum bicolor v3.1.1*, *Setaria viridis* v2.1 and *Oryza sativa v7.0* (obtained from Phytozome v. 12.1.6 [30]). Orthologs were used to retrieve functional annotations from *S. bicolor*, *S. viridis* and *Z. mays* downloaded from the Phytozome database.

### Gene expression analysis

To quantify transcript abundance the align_and_estimate_abundance Perl script of the Trinity package was applied, by mapping the reads of each biological replicate against the respective assembled transcriptome. In this analysis, SALMON was used as the abundance estimation method and quality check for biological replicates was assessed with the PtR utility from trinity. The Gene Expression Matrices were built using the abundance_estimates_to_matrix.pl script. Counts matrices were imported to R and the EdgeR package (v. 3.38.1) [42] was used for the Differential expression analysis. *P*-values were FDR corrected and genes with an FDR < 0.01 and a log2 fold change > 1 were considered as significant.

### Gene ontology enrichment analysis

The Gene Ontology (GO) enrichment analysis was performed using the R package TopGO v2.42 [43]. GO terms for each transcript were obtained from *S. bicolor*, *Z. mays* and *S. viridis* genomes from the phytozome database. P-values were adjusted using the “elim” algorithm. A term was considered significant if it had an adjusted *p* value < 0.01.

## Supplementary Information

**Additional file 1:  **Figures S1, S2, S3, S4, S5, S6 and S7. Tables S1 and S2.

**Additional file 2:** Results of PCA enrichment analyses.

**Additional file 3: **Results of Gene Ontology (GO) enrichment analyses.

**Additional file 4: **OG lists involved in photosynthetic and developmental processes.

## Data Availability

The Illumina RNA-Seq reads and transcriptomes assembly supporting the conclusions of this article are available from the NCBI Sequence Read Archive (PRJNA813546, https://dataview.ncbi.nlm.nih.gov/object/PRJNA813546?reviewer=6vmhgdsbsobv6pd6u6tr9rp4oe).

## Abbreviations

DEG: Differentially Expressed Genes
DEO: Differentially Expressed Orthogroups
GO: Gene Ontology
IBS: Inner Bundle Sheath Cell
M: Mesophyll Cell
OBS: Outer Bundle Sheath Cell
OG: Orthogroup
ORF: Open Reading Frame
VB: Vascular Bundle
