# Supplementary material for: De novo transcriptome assemblies of C3 and C4 non-model grass species reveal key differences in leaf development
Source: BMC Genomics. 2023 Feb 6;24:64. doi: 10.1186/s12864-022-08995-7 (PMC9901097; doi:10.1186/s12864-022-08995-7)
Supplement: Supplementary file 1 — Additional file 1: Figures S1, S2, S3, S4, S5, S6 and S7. Tables S1 and S2. [file 12864_2022_8995_MOESM1_ESM.docx]

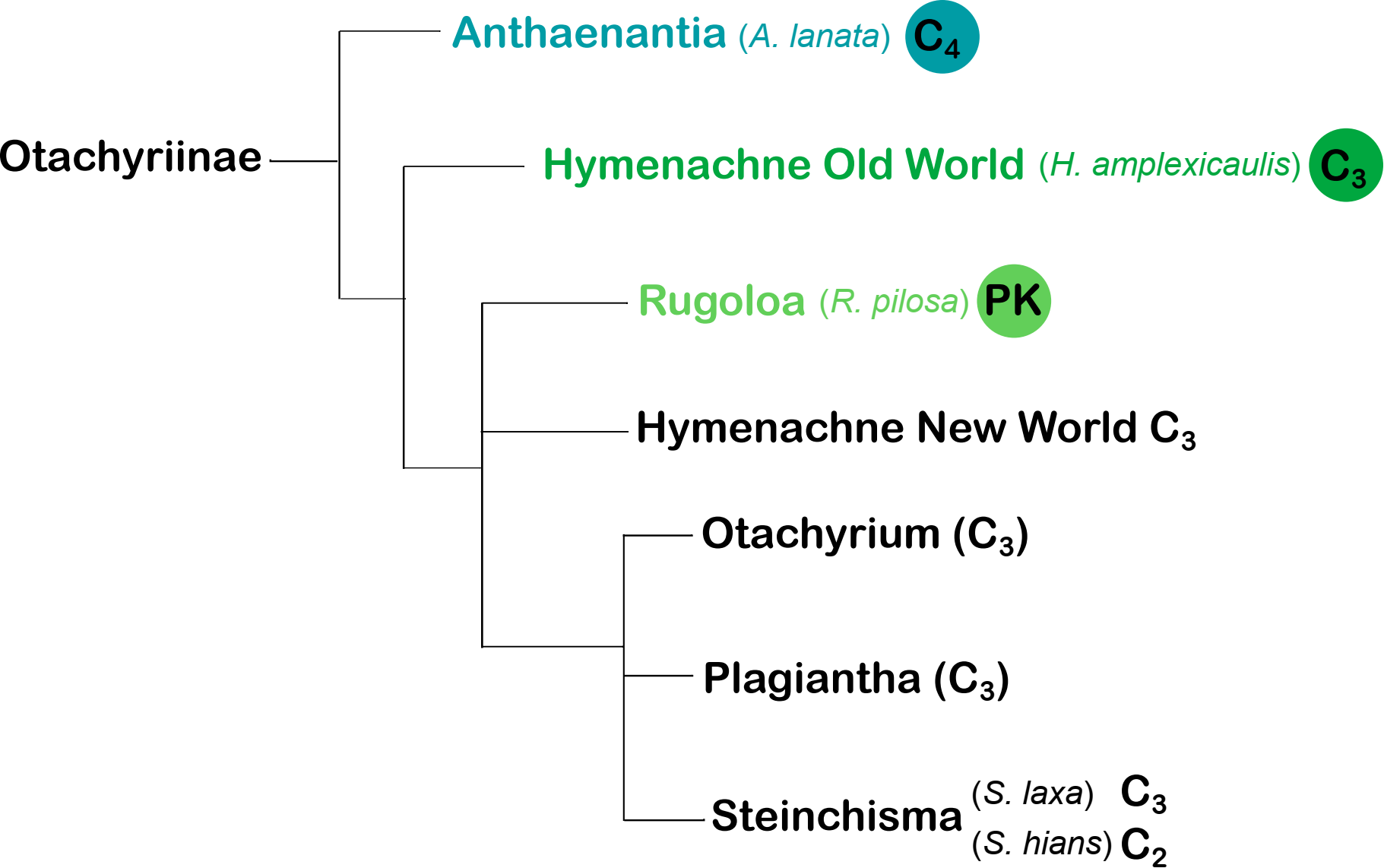


**Figure S1:** Otachyriinae lineage species tree modified from Acosta et al., 2019. In colors, the species used in this study.


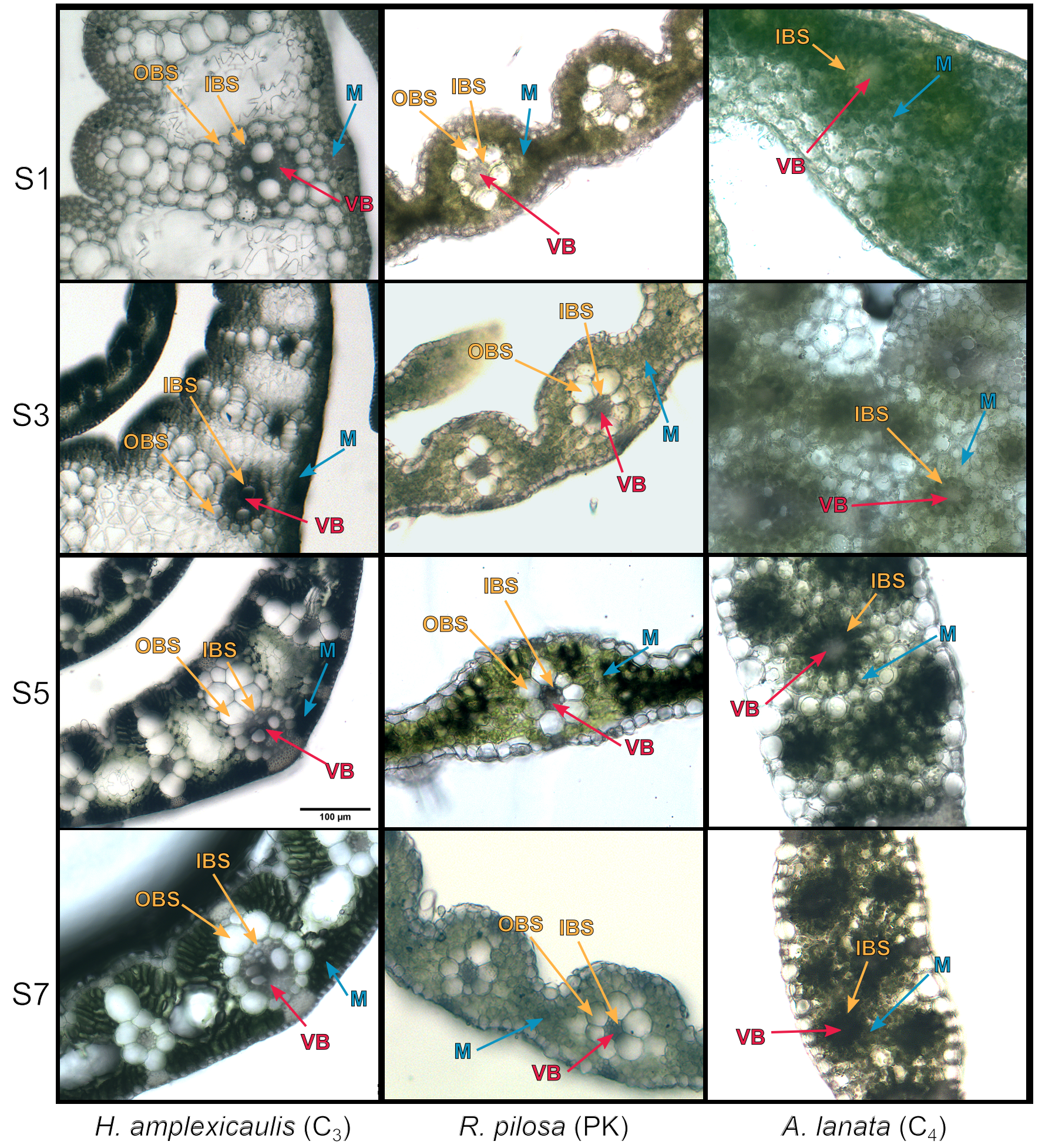


**Figure S2**: Sampling gradient in the 5^th^ leaf of three Otachyriinae subtribe species. Light microscope photographs of S1, S3, S5 and S5 cross section cuts from C_3_ *H. amplexicaulis*, PK *R. pilosa*, and C_4_ *A. lanata*.


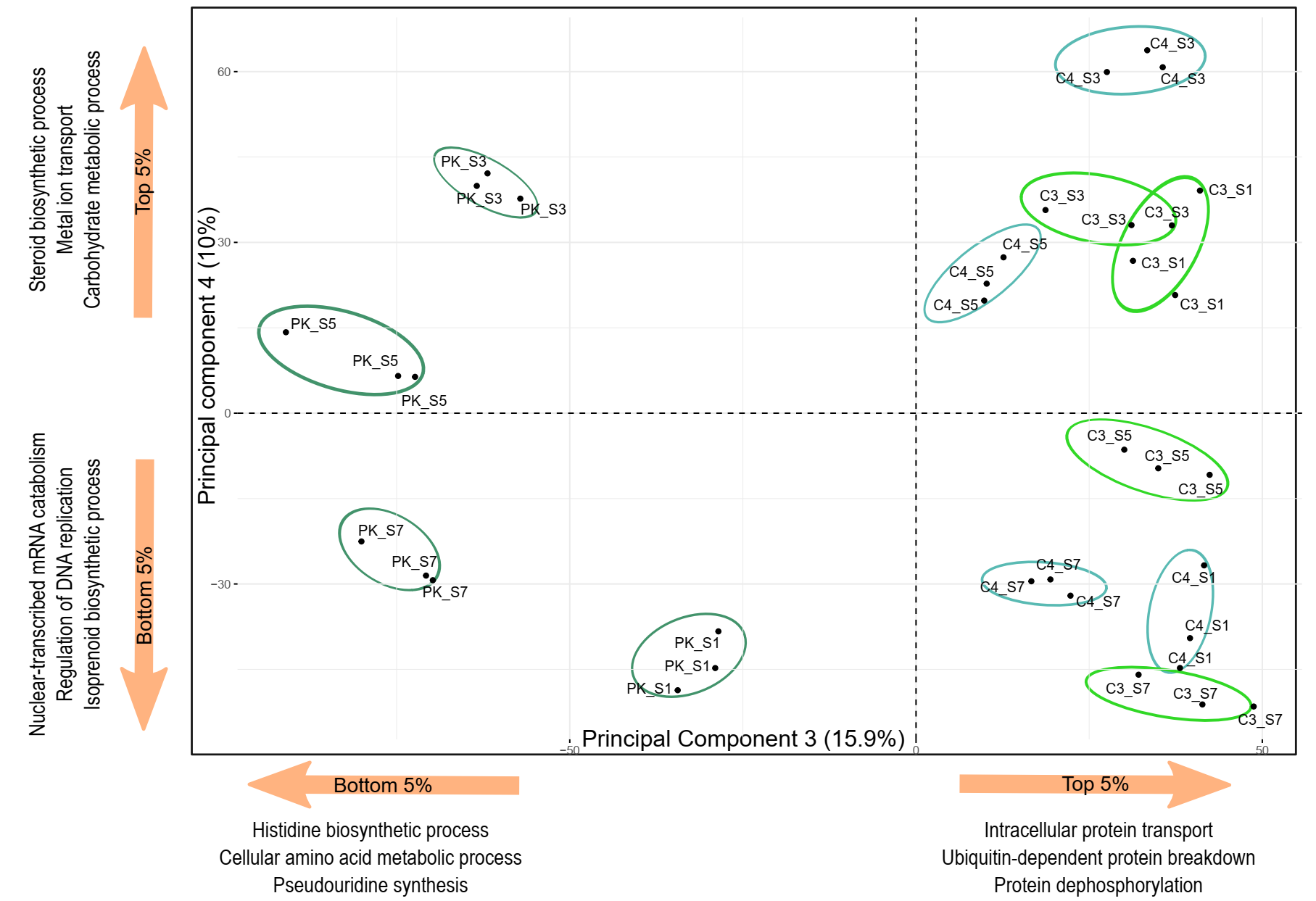


**Figure S3:** Principal components analysis of the samples (PCA3 vs PCA4). GO terms enriched in orthogroups with the highest load in each direction of each PC are indicated in the corresponding axis.


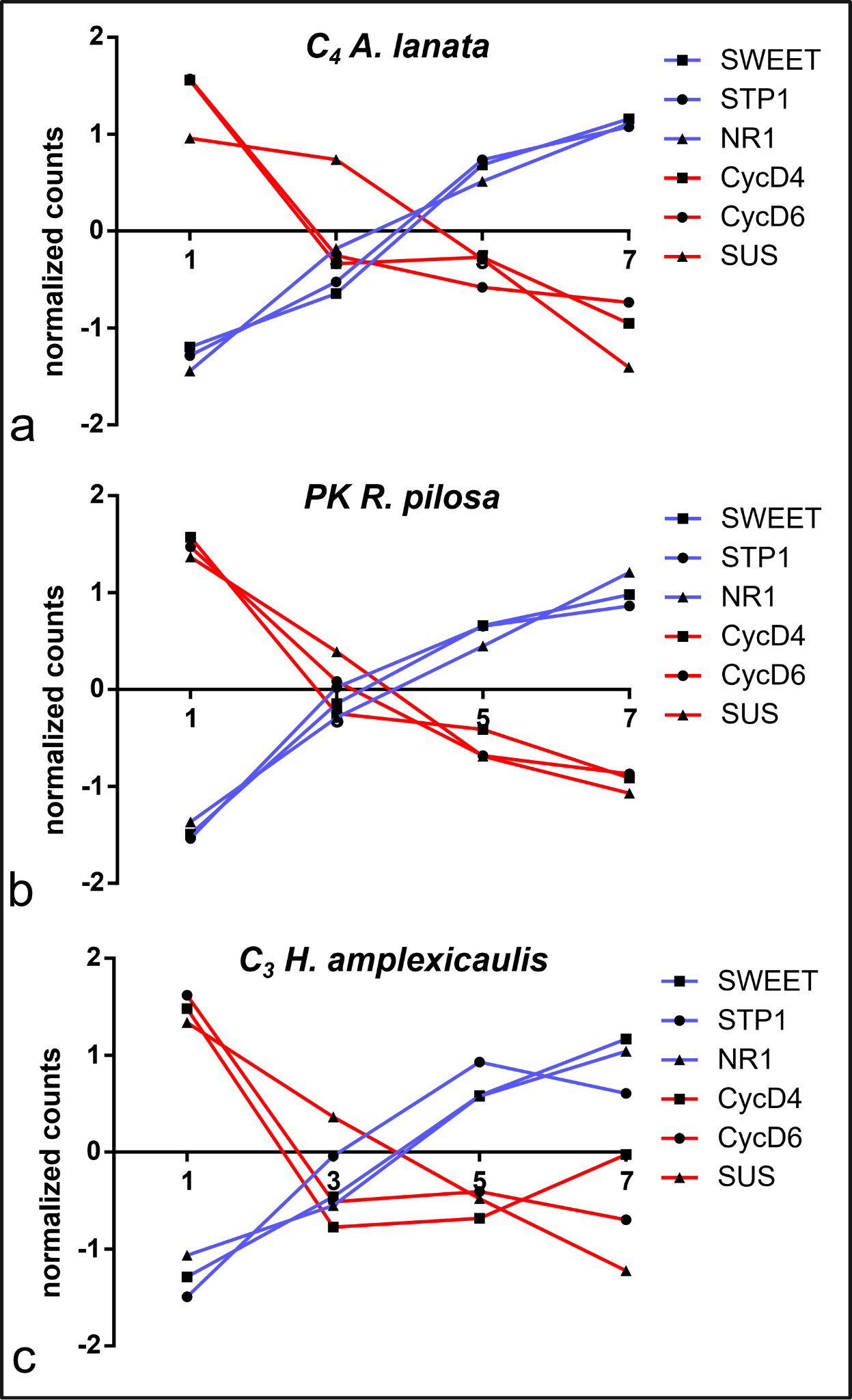


**Figure S4:** Relative expression levels for sink-source leaf markers C_4_ *A. lanata,* (a), PK *R. pilosa* (b) and C_3_ *H. amplexicaulis* (c). Abbreviation: CycD4: cell cycle modulator Cyclin D4. CycD6: cell cycle modulator Cyclin D6. NR1: nitrate reductase 1. SUS: sucrose synthase. SWEET: sucrose transporter SWEET. STP1: sucrose transporter STP1.

**
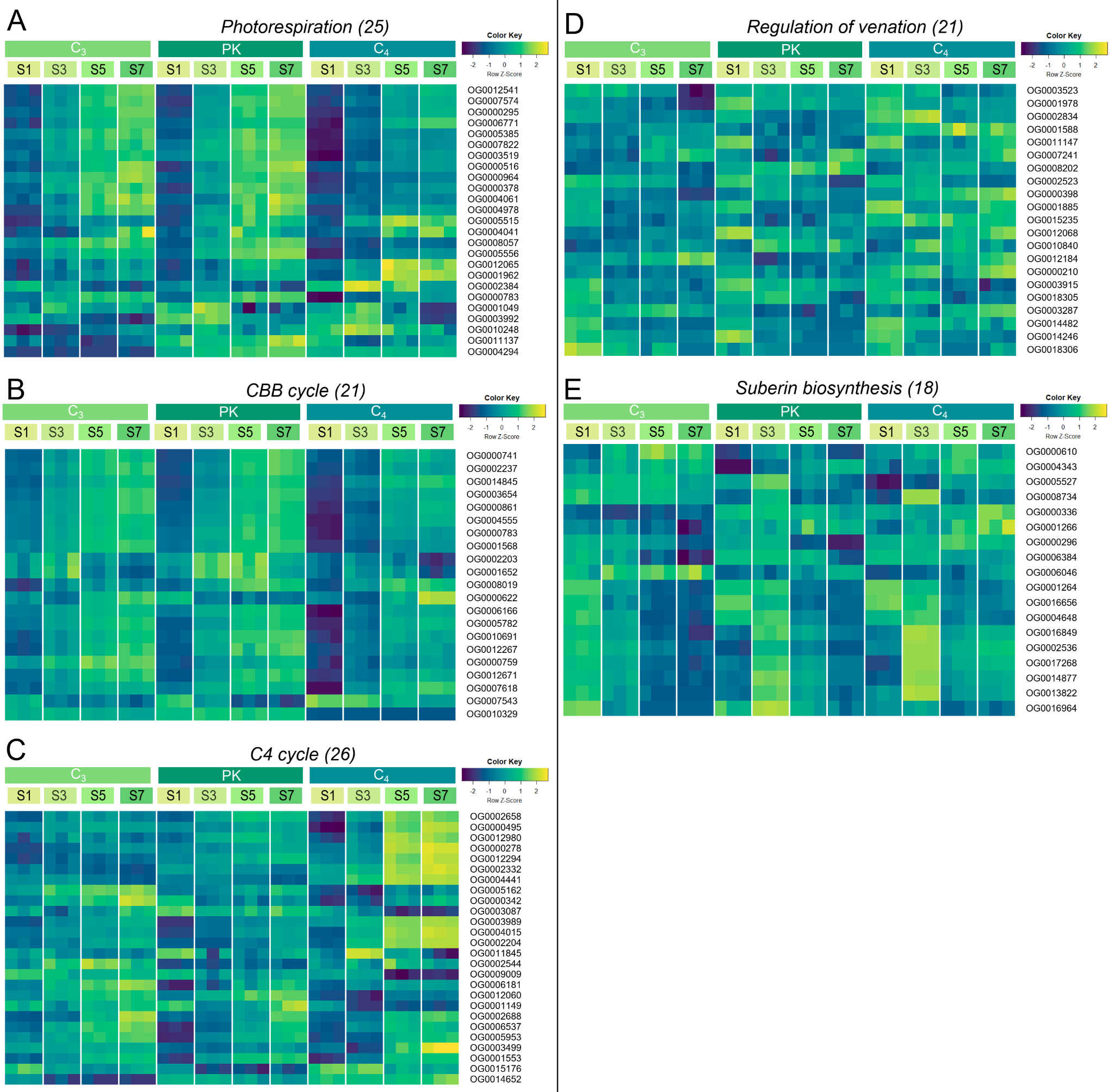
**

**Figure S5:** Heatmaps showing OG expression across leaf segments. (A) Photorespiration OG. (B) CBB cycle OG. (C) C_4_ cycle OG. (D) Regulation of venation OG. (E) Suberin biosynthesis OG.


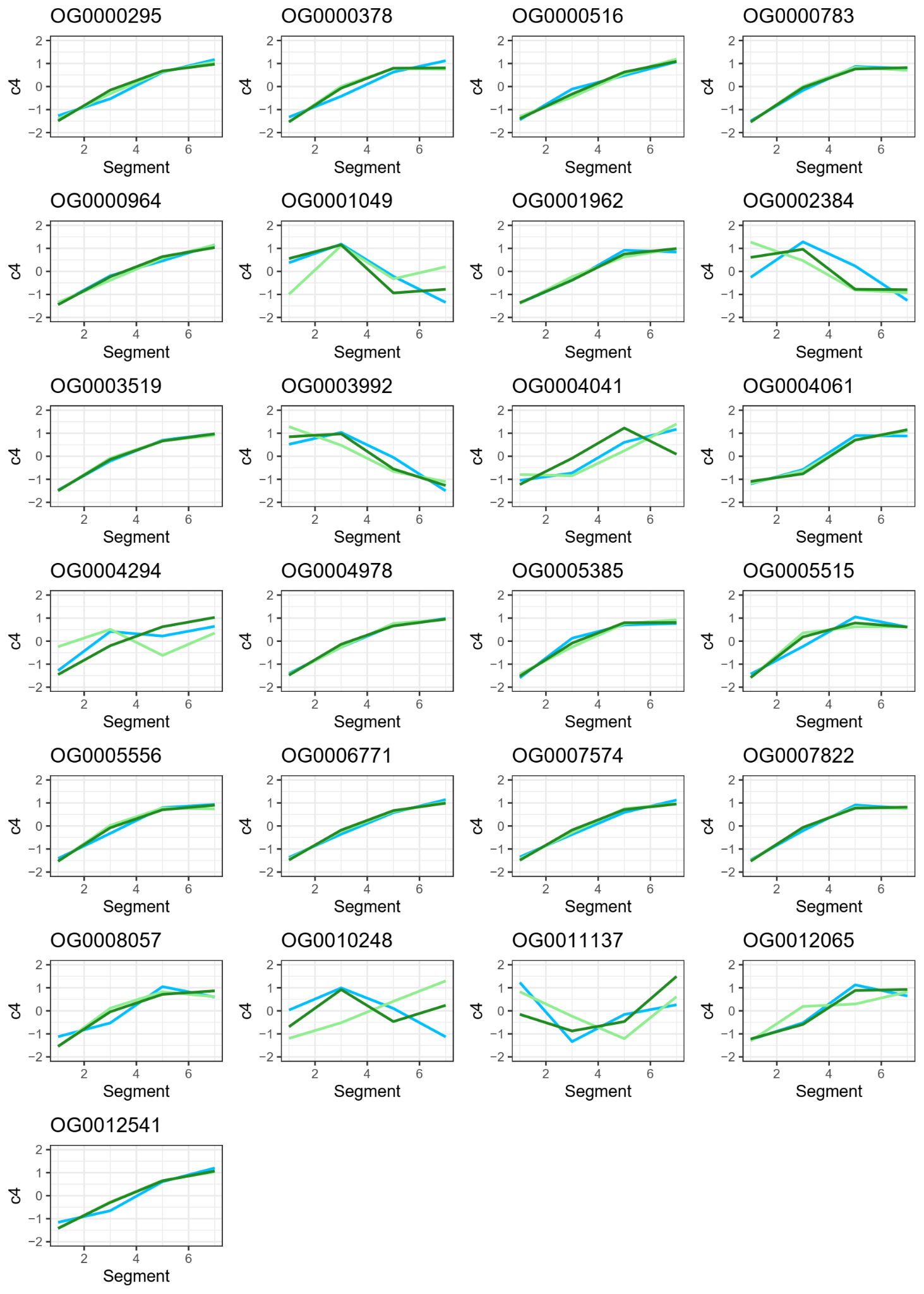


**Figure S6:** Expression patterns for photorespiration related OG. OG mean expression values from S1, 3, 5 and 7 for C3-H. amplexicaulis (light green), PK-R. pilosa (dark green) and C4-A. lanata (light blue). Relative expression (z-score normalization) across segments.


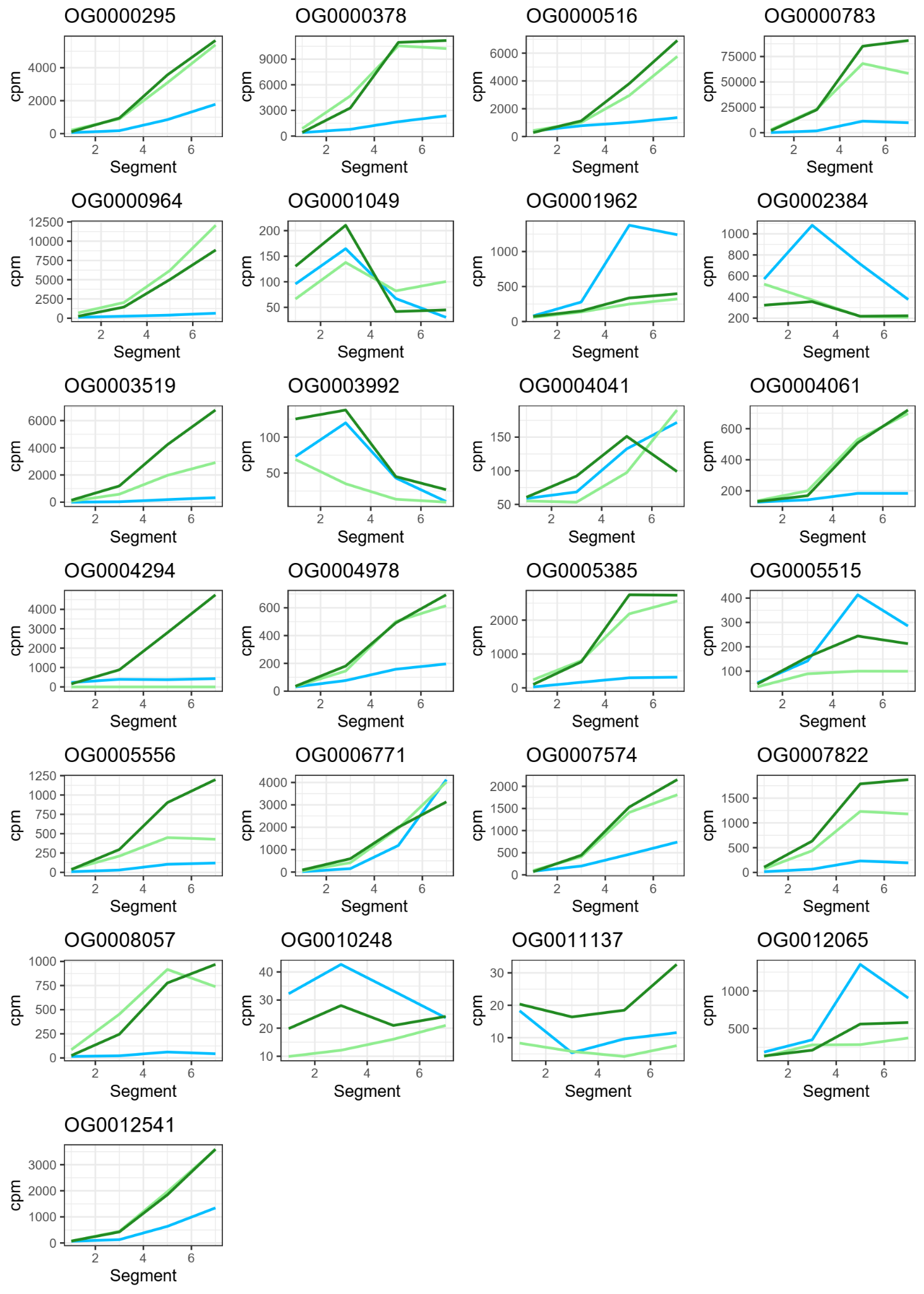


**Figure S7:** Expression patterns for photorespiration related OG. OG mean expression values from S1, 3, 5 and 7 for C3-H. amplexicaulis (light green), PK-R. pilosa (dark green) and C4-A. lanata (light blue). Count per million (cpm) across segments.

**Table S1**: Annotation statistics for C_3_ species *H. amplexicaulis*, PK species *R. pilosa* and C_4_ species *A. lanata* de novo transcriptome assemblies in each step of assembly.

| **De novo assembly (Trinity assembly)** | ***H. amplexicaulis (C_3_)*** | ***R. pilosa (PK)*** | ***A. lanata (C_4_)*** |
| --- | --- | --- | --- |
| transcripts | 245892 | 164363 | 355460 |
| genes | 157690 | 69387 | 167531 |
| SALMON mapping rate [%] | 97.5 | 96.9 | 95.2 |

| **Isoforms removed** | ***H. amplexicaulis (C_3_)*** | ***R. pilosa (PK)*** | ***A. lanata (C_4_)*** |
| --- | --- | --- | --- |
| transcripts | 157690 | 69387 | 167531 |
| genes | 157690 | 69387 | 167531 |
| SALMON mapping rate [%] | 90.6 | 90.7 | 87.0 |

| **No redundant transcriptome (after CD-HIT)** | ***H. amplexicaulis (C_3_)*** | ***R. pilosa (PK)*** | ***A. lanata (C_4_)*** |
| --- | --- | --- | --- |
| transcripts | 157361 | 69191 | 165254 |
| genes | 157361 | 69191 | 165254 |
| SALMON mapping rate [%] | 90.6 | 90.7 | 87.0 |

| **Final transcriptome (after Transdecoder)** | ***H. amplexicaulis (C_3_)*** | ***R. pilosa (PK)*** | ***A. lanata (C_4_)*** |
| --- | --- | --- | --- |
| transcripts | 56064 | 29370 | 50890 |
| genes | 56064 | 29370 | 50890 |
| SALMON mapping rate [%] | 62.7 | 61.6 | 55.9 |

**Table S2**: Annotation statistics for the *H. amplexicaulis*, *R. pilosa* and *A. lanata* de novo transcriptome assemblies before and after filtering low expressed transcripts (transcripts with at least 1 cpm in 3 replicates libraries were kept).

|  | ***H. amplexicaulis*** | ***R. pilosa*** | ***A. lanata*** |
| --- | --- | --- | --- |
| **N° of protein coding transcripts** | 56064 | 29370 | 50890 |
| **N° of transcripts in Orthogroups** | 43331 [77.3%] | 25727 [87.6%] | 38863 [76.4%] |
| **N° of transcripts annotated** | 25134 [44.8%] | 21710 [73.9%] | 27147 [53.3%] |

|  | ***H. amplexicaulis*** | ***R. pilosa*** | ***A. lanata*** |
| --- | --- | --- | --- |
| **N° of protein coding transcripts** | 19682 | 18928 | 22788 |
| **N° of transcripts in Orthogroups** | 19033 [96.7%] | 18410 [97.3%] | 21088 [92.6%] |
| **N° of transcripts annotated** | 16856 [85.6%] | 17091 [90.3%] | 18162 [79.7%] |
